# Is surgeon annual case volume related with intra and postoperative complications after ventral hernia repair? Uni- and multivariate analysis of prospective registry-based data

**DOI:** 10.1007/s10029-024-03129-2

**Published:** 2024-08-07

**Authors:** R. van den Berg, F. P. J. den Hartog, A. G. Menon, P. J. Tanis, J. F. Gillion, The Hernia Club members

**Affiliations:** 1 Department of Surgery, Erasmus University Medical Center, Dr. Molewaterplein 40, 3015 GD Rotterdam, The Netherlands; 2 Department of Surgery, IJsselland Hospital, Capelle Aan Den IJssel, The Netherlands; 3 Hôpital Privé d’Antony, 1 Rue Velpeau, 92160 Antony, France

**Keywords:** abdominal wall, incisional hernia, parastomal hernia, primary ventral hernia, surgical experience, annual operating volume

## Abstract

**Background:**

Literature on a potential relationship between surgeon case volume and intra- or postoperative complications after ventral hernia repair remains scarce.

**Methods:**

Patients who underwent ventral hernia repair between 2011 and 2023 were selected from the prospectively maintained French Hernia-Club Registry. Outcome variables were: intraoperative events, postoperative general complications, surgical site occurrences, surgical site infections, length of intensive care unit (ICU), and patient-reported scar bulging during follow-up. Surgeons’ annual case volume was categorized as 1–5, 6–50, 51–100, 101–125, and > 125 cases, and its association with outcome volume was evaluated using uni- and multivariable analyses.

**Results:**

Over the study period, 199 titular or temporary members registered 15,332 ventral hernia repairs, including 7869 primary, 6173 incisional, and 212 parastomal hernia repairs. In univariate analysis, surgeons’ annual case volume was significantly related with all the postoperative studied outcomes. After multivariate regression analysis, annual case volume remained significantly associated with intra-operative complications, postoperative general complications and length of ICU stay. A primary repair was independently associated with fewer intra- and post-operative complications.

**Conclusion:**

In the present multivariable analysis of a large registry on ventral hernia repairs, higher surgeon annual case volume was significantly related with fewer postoperative general complications and a shorter length of stay, but not with fewer surgical site occurrences, nor with less patient-reported scar bulging. Factors in the surgeons’ case mix such as the type of hernia have significant impact on complication rates.

**Supplementary Information:**

The online version contains supplementary material available at 10.1007/s10029-024-03129-2.

## Introduction

Ventral abdominal wall hernias are a very common condition with approximately 350.000 ventral hernia repair operations per year in the United States of America alone, with an estimated annual financial impact of 3.2 billion dollars [1]. Non-inguinal, ventral hernias mainly encompass primary (PVH) [2], incisional (IH) [3] and parastomal hernias (PSH) [4]. IH is a common complication after abdominal surgery with incidence rates reaching levels of up to 60% in high-risk patients [5–7]. PVHs occur spontaneously or congenitally in up to approximately 20% of healthy adults [8]. Examples of PVHs are umbilical and epigastric hernias. These hernias can cause discomfort, pain, and an impaired quality of life [9]. In the worst case, intestinal incarceration can occur, with intestinal ischemia as a possible outcome. If an incarceration cannot be manually reversed, patients have to undergo emergency surgery, possibly resulting in an intestinal resection [10]. PSH is the most frequent complication following colostomy or ileostomy with an incidence of up to 50%. PSH can result in impaired stoma function and the need for stoma revision [10, 11].

The only curative therapy for any type of these hernias is surgery. The complexity of hernia surgery depends on patient-related factors (e.g., age, BMI, comorbidities, smoking and ASA score), hernia-related factors (e.g., defect size, defect location, whether it is a recurrence and contamination grade) and technical difficulties increasing from primary to incisional and parastomal hernia repairs [12]. Ventral hernia repair has become more complex [13] as surgeon skill-demanding techniques have been recently developed [3, 14–17], concurrently with the development of multimodal prehabilitation [18].

Thus, both the hospital caseload (roughly estimating the experience of the whole surgical team) and the surgeon case volume (roughly estimating the surgeon’s experience/skills) were logically developed as quality indicators [19, 20], in rectal [21], upper GI [22], intra-abdominal emergency surgery [23], and also in hernia repair surgery.

In abdominal wall hernia repairs, aside from studies investigating on the surgeon learning curve, which differs for every procedure, the surgeon case volume [24, 25] and the hospital caseload [26, 27] were mainly investigated in groin hernia repair. In ventral hernia repairs, studies are even rarer, investigating on hospital caseload [28], or surgeon case volume [29, 30]. The data of these studies, based on state-wide or nationwide administrative records, are steadily comprehensive (e.g., reoperation rate for recurrence) but with a low granularity, due to a lack of medical variables systematically registered. Such medical variables are registered in our Club-Hernie registry allowing for multivariate analysis.

Thus, the aim of the present study was to investigate in a univariate and multivariate analysis on the relationship between the surgeon annual case volume and the postoperative outcomes after ventral hernia repair.

## Methods

This retrospective cohort study was conducted following the STROBE [31], the STROCSS statements [32], and the recommendations of the European Registry of Abdominal Wall Hernias working group [33].

### Study design

A uni- and multivariate analysis was retrospectively conducted, on the prospectively maintained data of the French Club-Hernie (CH) registry. All adult patients who underwent ventral hernia repair between 2011 and 2023 were selected from the registry. The annual case volume of the surgeons, according to their data entries, was used for the analysis of the outcomes.

### Hernia-club registry

The registry complies with the General Data Protection Regulation and is approved by the French ‘*Commission Nationale de l'Informatique et des Libertés’* (CNIL) (registration number: 1993959v0). The study's registry-based design, which guarantees that all data are anonymous and de-identified, collected with a patient ‘non-opposition’ agreement, complies with the national ethical standards of France and the Netherlands.

Titular CH members, surgeons specially interested in abdominal wall surgery, coopted by their peers, must acknowledge, and sign the charter of quality, which states that “all input must be registered in a consecutive, unselected and comprehensive manner and in real time”. Since 2011, founding members have been progressively replaced with younger members. The registry may also host temporary members such as participants in academic multicenter studies [34], surgeons validating the self-evaluating requirements of their professional certification (https://accreditation-des-medecins.fr/siam), and surgeons registering one or a few cases in order to be discussed in the national online monthly peer counselling meeting on complex abdominal wall cases.

### Data entries and follow-up modalities used in the hernia-club registry

CH members register the pre-, intra-, and 30-day postoperative data in the online database. Data entry is completed during the systematic 1st month visit scheduled with the operating surgeon. Afterwards, the dedicated CH clinical research assistant, independent from the surgical teams, manages the 2–5-year follow-up of the patients, following a formatted phone questionnaire including: late outcomes such as rehospitalizations, reoperations and their causes, confirmed recurrences (reoperated, TDM/ultrasound, and/or surgeon visit), suspected recurrences (localized bulging and/or local pain) late abscesses, chronic sinuses, and other late complications (e.g., bowel obstructions), PROM (patient related outcome measures) and quality of life. In case of any deviation from the normal course, a visit at the surgeon office is strongly recommended. The surgeon uses the surgeon dedicated tabs to complete the follow-up data.

After five unsuccessful attempts to get in touch with the patient at various times and dates, they are deemed lost to follow-up.

### Data extraction and variables used for the present study

Relevant baseline, surgical, and functional outcome variables were extracted from the Hernia-Club database. The Club Hernie registry data set was built to be compatible with the European Hernia Society (EHS) classification of abdominal wall hernias [35].

Extracted baseline variables comprised: age, gender, body mass index (BMI), ASA classification, diabetes mellitus, hernia recurrences, smoking status, emergency surgery, synchronous repair of multiple defects, wound classification (clean, clean-contaminated, contaminated, dirty), defect location and width, open or laparoscopic route, mesh site (intraperitoneal, sublay, subcutaneous) [18], and surgical operative time.

The intra-operative complications were defined as one or more of the following complications: bladder injury, bowel injury, severe bleeding, or general complication occurring during the procedure.

The postoperative complications were clustered as follows: i. General complications including isolated or combined medical complications such as heart attack, thrombophlebitis with or without pulmonary embolism, compartmental syndrome, neurological, arrhythmia, urinary retention, injection site inflammation within 30 days after surgery; ii. Surgical site infection (SSI) including all wound infections individualized in peri- (deep) or not peri-prosthetic (superficial) infected collections, and surgical site occurrence (SSO) including all peri- or not periprosthetic non infected collections; iii. Organ space (surgical) complication including intraperitoneal bleeding, peritonitis, bowel obstruction, and immediate recurrence; iiii. Combined complications including either one type of postoperative or surgical complication. In case of concurrent complications, the Clavien-Dindo [36] grading was based on the worse complication.

### Surgeon annual case volume

The annual surgeon case volume was defined as the annual number of their registered cases in the database, first considered as a continuous variable in scatter plot graphs and further as a categorized variable in uni- and multi-variate analysis. As no consensus about the threshold arose from the literature [37], we, before starting the study, arbitrarily have chosen five activity levels: C1 (1–5 cases); C2 (6–50 cases); C3 (51–100 cases); C4 (101–125 cases); C5 (> 125 cases).

### Outcomes of interest

Intra- and postoperative (general, surgical site, organ space) complications as previously described, ICU stay, and patient reported scar bulging identified during the follow-up formatted phone questionnaires.

### Statistical analysis

#### Missing data/imputations

Missing data entries (supplement material) were assumed to be missing at random (i.e., not related to the outcomes or study groups). Multiple imputations (30 iterations and 10 imputations) were performed to allow the use of all available data (Suppl. B).

#### Univariate analysis

The effect of the surgeons annual volume was assessed in univariate logistic and negative binomial regression models. The effect of the surgeons annual volume was reported with odds-ratios (OR) or incidence-rate ratios (IRR), confidence intervals (CI) and p-values.

#### Multivariate analysis

Confounding factors were controlled in a multivariate logistic regression for the dichotomous outcomes and a negative binomial regression for the continuous (right skewed count-data) outcomes.

Discrete variables were presented as absolute numbers with percentages. Continuous variables were presented as median and interquartile range (IQR). The distribution of the variables was assessed graphically. Discrete variables were compared using the Chi-square test and continuous variables were compared either using Student’s T test or the Mann–Whitney U test as appropriate (i.e., normality was assessed graphically in quantile–quantile plot).

For the dichotomous outcomes, the multivariable logistic regression included the following factors: age, gender, BMI, undernutrition, active smoking, ASA score, history of hernias (ventral hernia in other location, groin or hiatal hernia, aortic aneurysm surgery), healing factors (anticoagulants, chemotherapy or immunosuppression, radiotherapy, diabetes, steroids), dissection factors (extraperitoneal previous dissection, such as a patients that had a history of pelvic lymph node dissection), complicated hernia (severe pain, strangulated hernia, bowel obstruction, cellulitis), Altemeier wound classification, EHS classification, type of repaired hernia (primary, incisional or parastomal), the use of component separation and the mesh site. For the continuous outcomes, the distributions of the outcome parameter were assessed graphically, and negative binomial regression was used. The same factors corrected for in the logistic regression models were used in the negative binomial models. A p value smaller than 0.05 was considered significant. The statistical analysis was performed using R, version 4.4.1 (21).

## Results

### Patient characteristics

The characteristics of the included patients are available in supplementary materials (Suppl. A).

### Surgeon annual case volume

Over the study period, 199 titular or temporary members registered 15,332 ventral hernia repairs in the CH registry, including 7,869 primary ventral hernia repairs (PVHR), 6,173 incisional ventral hernia repairs (IVHR), and 212 parastomal hernia repairs (PSHR). In 94% of cases, the operation was conducted by a single surgeon. The median (IQR) of their registered cases was 70 (44–85). The categorized distribution of surgeons according to their annual registered case volume was: C1 (1–5 cases): 81 surgeons; C2 (6–50 cases): 86 surgeons; C3 (51–100 cases): 28 surgeons; C4 (101–125 cases): 3 surgeons; C5 (> 125 cases): 1 surgeon. The distribution of the surgeons, according to their annual registered case volume, is graphically shown in the supplementary files (Suppl. B). Emergency surgery and incarcerated hernias were evenly distributed throughout the annual case volume groups (Suppl. H). Since the annual volume of the surgeons stayed relatively constant through the years that the surgeons included patients into the database, the year with the maximum number of registered cases was taken as the annual volume for the surgeon.

**Fig. 1:**
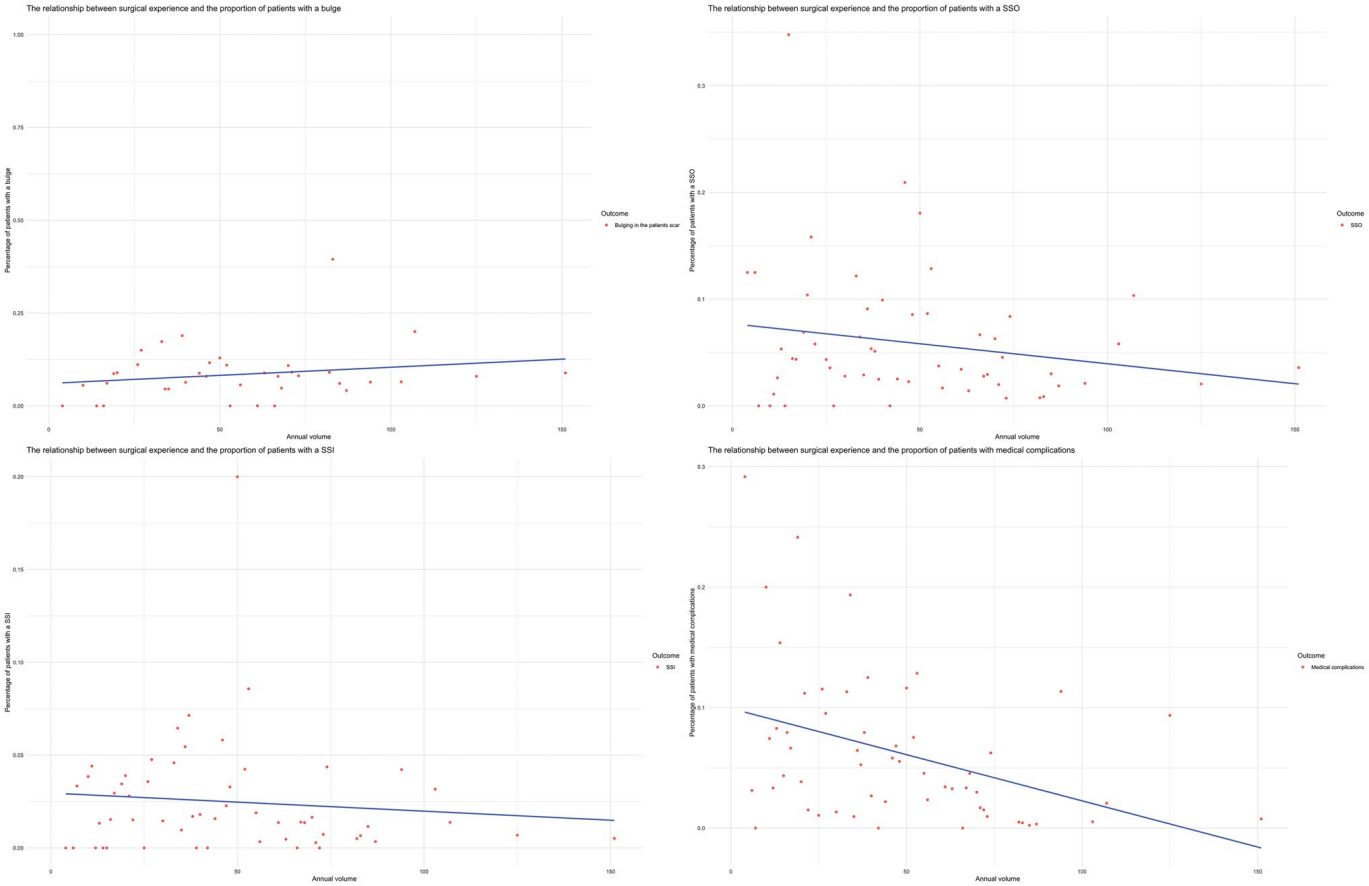
Relationship between the annual case volume categorized and different types of complications

Graphically, the scatter graphs with tendency lines (Fig. 1) show the correlation of annual case volume and the mean percentage of the outcomes per operation. A downwards sloping line can be seen on every outcome except for the patient reported scar bulging. Similarly, the tendency line of the mean ICU decreases as the surgeon case volume increases (Fig. 4).

**Fig. 2:**
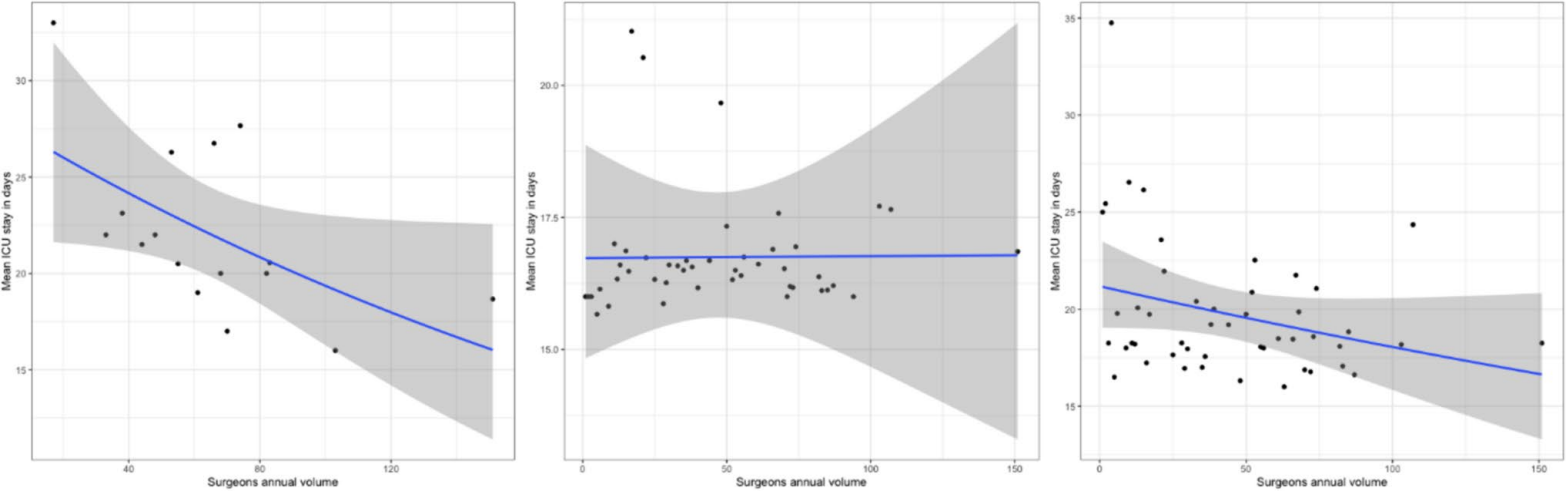
The trend of ICU stay in the different types of hernia repair set out against the annual volume. From left to right: PSHR, PVHR, IVHR

**Fig. 3:**
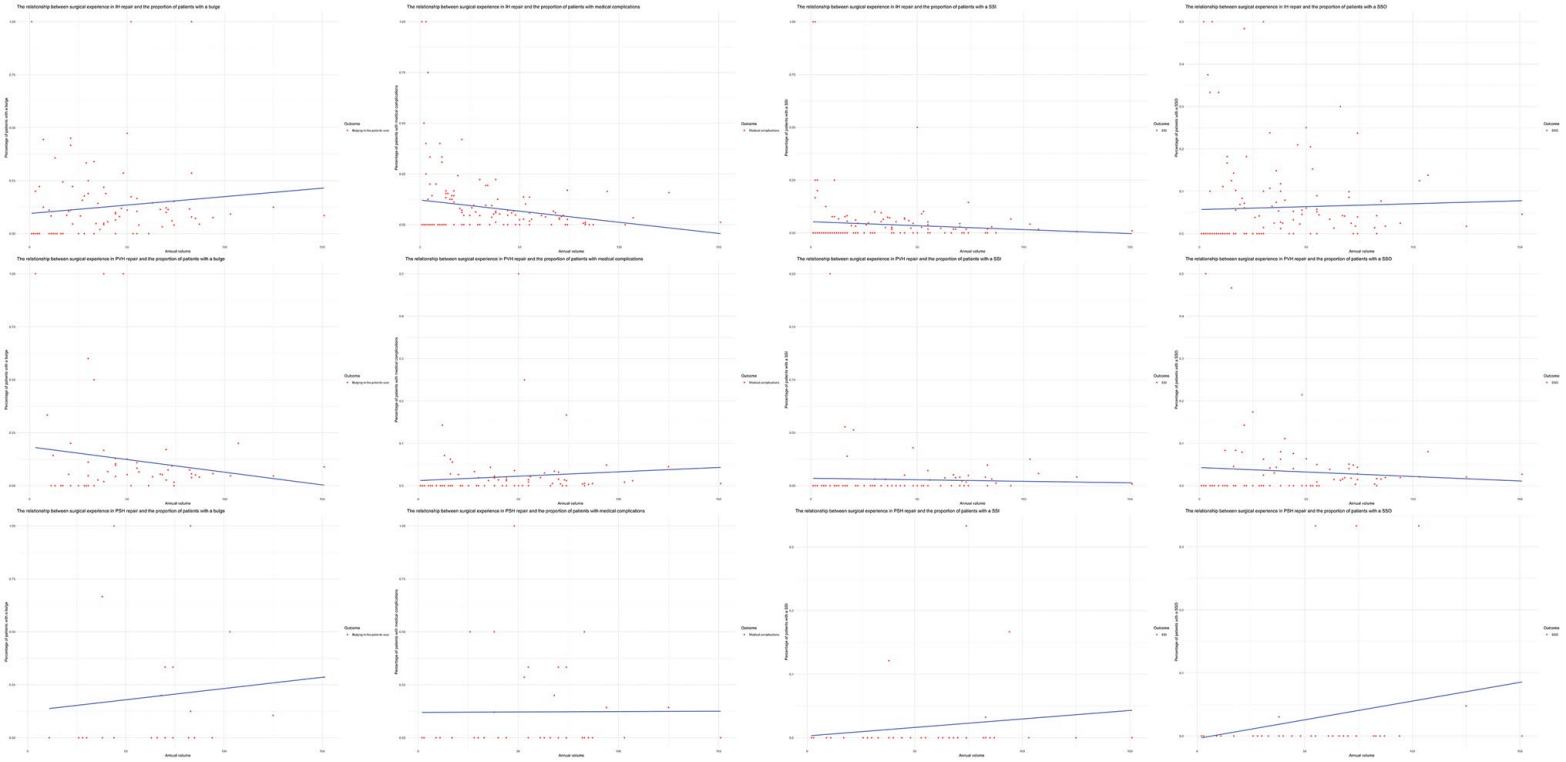
Relationship between the annual case volume categorized and different types of complications stratified for IVHR, PSHR, and PVHR

Furthermore, different trends for the outcomes were found on the various types of hernia repair surgery: IVHR, PVHR, PSHR (Fig. 3). For the ICU stay, a trend towards less ICU stay can be seen with a higher annual volume in the IVHR and PSHR groups but no difference can be seen in the PVHR group (Fig. 2).

**Fig. 4:**
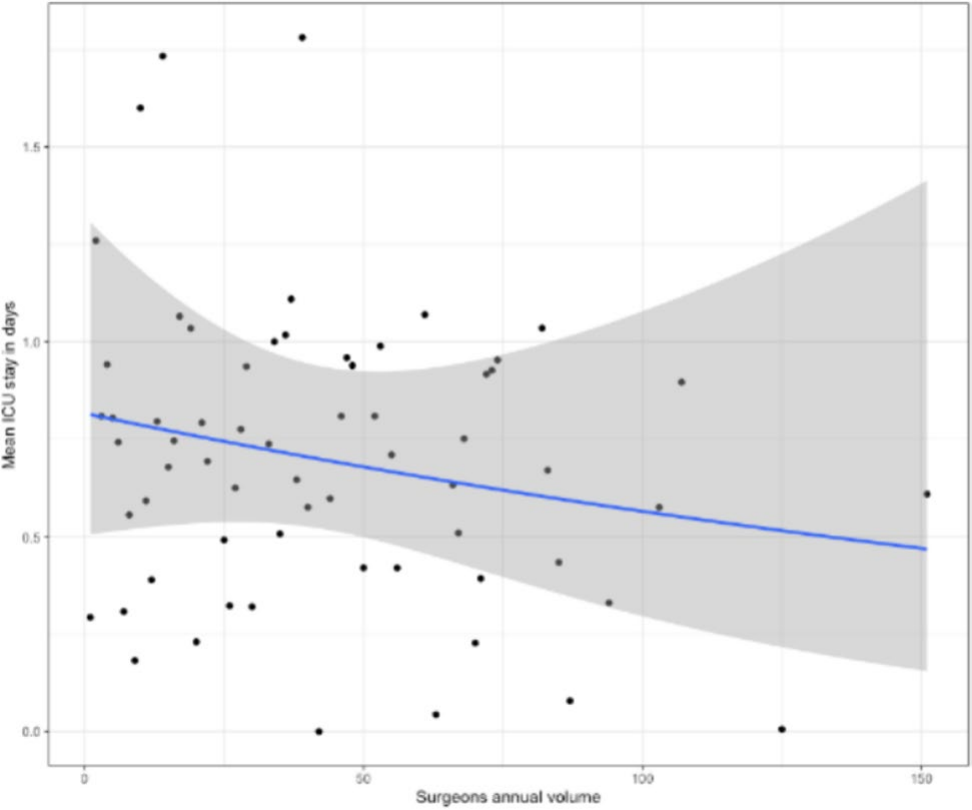
Relationship with tendency line between annual case volume and the average ICU length of stay of the patients for every individual surgeon

### Univariate analysis

The univariate analysis revealed that all investigated outcomes, with the exception of patient-reported scar bulging, exhibited a significant correlation with the annual volume of surgeries conducted by the surgeon (Suppl. G). Specifically, the incidence of both intra- and postoperative complications declined in conjunction with an augmentation in the number of cases performed by the surgeon. Additionally, as demonstrated in Suppl. H, there was a notable reduction in the length of ICU stay as the surgeon's activity increased. This reduction was at its peak in the C5 group with an IRR of 0.67 (95%CI 0.61–0.75).

### Multivariate analysis

The multivariate analysis revealed that all investigated outcomes, with the exception of patient-reported scar bulging and the occurrence of an SSI, exhibited a significant correlation with the annual volume of surgeries conducted by the surgeon (Table 1). The full multivariate analysis can be found in the supplementary files (Suppl. C).

**Table 1:** Multivariable logistic regression with binary outcome measures

Multivariate regression analysis																				
Characteristic	SSI				SSO				30 Day Medical Complications				Feel a Bulge				Intra-Operative Complications			
	N	OR^a^	95% CI^a^	p-value	N	OR^a^	95% CI^a^	p-value	N	OR^a^	95% CI^a^	p-value	N	OR^a^	95% CI^a^	p-value	N	OR^a^	95% CI^a^	p-value
Peak annual volume^b^																				
C1	126	–	–		126	–	–		125	–	–		64	–	–		133	–	–	
C2	3,506	0.73	0.30, 1.77	0.5	3,506	1.00	0.50, 1.98	> 0.9	3,530	0.52	0.29, 0.93	**0.029**	1,886	1.47	0.61, 3.51	0.4	3,621	0.64	0.26, 1.55	0.3
C3	6,866	0.63	0.26, 1.53	0.3	6,866	0.76	0.38, 1.50	0.4	6,902	0.34	0.19, 0.62	** < 0.001**	3,955	1.02	0.42, 2.44	> 0.9	7,513	0.47	0.19, 1.13	0.091
C4	961	0.60	0.22, 1.64	0.3	961	1.07	0.51, 2.24	0.9	962	0.51	0.26, 0.98	**0.044**	567	0.87	0.35, 2.22	0.8	967	0.53	0.19, 1.47	0.2
C5	1,171	0.21	0.06, 0.71	**0.012**	1,171	0.72	0.34, 1.52	0.4	1,172	0.08	0.03, 0.19	** < 0.001**	652	1.16	0.47, 2.89	0.7	1,171	0.15	0.04, 0.53	**0.003**

^a^*OR* odds ratio, *CI* confidence interval

^b^Peak annual volume was categorized into C1 (1–5 cases); C2 (6–50 cases); C3 (51–100 cases); C4 (101–125 cases); C5 (> 125 cases)

Bold values denote statistical significance at the p < 0.05 level

### Surgical site infection

Out of 12,630 documented cases, 223 (1.77%) SSIs were reported. A higher annual volume was significantly related with less SSIs in the C5 group (OR 0.21; 95%CI 0.06–0.71)) compared to the C1 group as seen in Table 1.

### Surgical site occurrence

Out of 12,630 documented cases, 527 (4.17%) SSOs were reported. A higher annual volume was not significantly related with less SSOs in any of the experience groups.

### Thirty-day medical complications

Out of 12,691 documented cases single or combined medical complications occurred in 449 patients (3.54%). Surgeon annual case volume was significantly related to the 30-day medical complications throughout all groups with the largest effect seen in group C5 (OR 0.08; 95%CI 0.03–0.19) as seen in Table 1.

### Patient-reported scar bulging

Out of 7123 documented cases 620 (8.70%) patients reported a scar bulging. A higher annual volume was not significantly related with less patient-reported scar bulging in any of the different groups as seen in Table 1.

### Intra-operative complications

Out of 13,405 documented cases, 214 (1.60%) intraoperative events were reported. The surgeon annual case volume was significantly related to intraoperative complications in the C5 group having significantly lower amounts of intraoperative complications (OR 0.15; 95%CI 0.04–0.53) as seen in Table 1.

### Combined intra- and post-operative complications

Combining all the intra- and post-operative complications, resulted in 1576 (22.6%) complications in 6975 patients. The annual volume of the surgeon was significantly related to less complications across all groups. The largest effect size was noted in the C5 group compared to the C1 group (OR 0.10; 95% CI 0.05–0.21) (Suppl. E).

### ICU stay

The duration of ICU stay, completed in 8016 cases, was significantly (p < 0.001) related to the surgeon annual case volume. Across all groups C2 through C5 a significant effect was noted when compared with the C1 group. The IRR of all the groups had the same effect size of around 0.75 (Table 2). The full multivariate analysis on ICU stay can be found in the supplementary files (Suppl. D).

**Table 2:** Multivariable negative-binomial regression on ICU stay

Characteristic	ICU stay			
	N	IRR^a^	95% CI^a^	p-value
Peak annual volume^b^				
C1	114	–	–	
C2	2514	0.76	0.65, 0.89	** < 0.001**
C3	4322	0.78	0.66, 0.91	**0.001**
C4	388	0.72	0.60, 0.86	** < 0.001**
C5	678	0.75	0.63, 0.89	** < 0.001**

^*a*^*IRR* incidence rate ratio, *CI* confidence interval

^b^Peak annual volume was categorized into C1 (1–5 cases); C2 (6–50 cases); C3 (51–100 cases); C4 (101–125 cases); C5 (> 125 cases)

Bold values denote statistical significance at the p < 0.05 level

Among the other variables (Suppl. C) significantly related to less combined per- and post-operative complications was: undergoing PVHR or PSHR in comparison to IVHR. Across all groups significantly less complications were seen in the PVHR group and for the PSHR group a lower amount of SSO was seen compared to IVHR (Table 3).

**Table 3:** Effect of IVHR, PSHR and PVHR on the binary outcome measures in multivariable logistic regression

Regression analysis																				
Characteristic	Table SSI				Table SSO				Table 30 Day Medical Complications				Table Feel a Bulge				Table Intra-Operative Complications			
	N	OR^a^	95% CI^a^	p-value	N	OR^a^	95% CI^a^	p-value	N	OR^a^	95% CI^a^	p-value	N	OR^a^	95% CI^a^	p-value	N	OR^a^	95% CI^a^	p-value
Type of repaired hernia																				
IVHR (incisional ventral hernia repair)	5,519	–	–		5,519	–	–		5,541	–	–		3,128	–	–		5,829	–	–	
PSHR (parastomal hernia repair)	180	0.45	0.18, 1.10	0.078	180	0.32	0.13, 0.82	**0.018**	184	0.95	0.53, 1.69	0.9	85	1.73	0.85, 3.50	0.13	192	0.69	0.33, 1.43	0.3
PVHR (primary ventral hernia repair)	6,931	0.52	0.33, 0.81	**0.004**	6,931	0.76	0.59, 0.99	**0.039**	6,966	0.43	0.32, 0.58	** < 0.001**	3,911	0.69	0.53, 0.88	**0.003**	7,384	0.46	0.29, 0.71	** < 0.001**

^a^*OR* odds ratio, *CI* confidence interval

Bold values denote statistical significance at the p < 0.05 level

## Discussion

In our univariate and multivariate analysis of 15,332 ventral hernia repairs collected in a prospectively maintained registry, we found that the annual surgeon case volume is an independent and significant predictive factor for the development of intraoperative events, 30-day postoperative complications, and ICU stay in hernia repair surgery.

In ventral hernia repairs, studies investigating on hospital caseload [28], or surgeon case volume [29, 30] are, to our best knowledge, scarce and mainly based on administrative records. Their highest interest hinges on the comprehensiveness of the administrative data such as hospital caseload, surgeon case volume, hospital length of stay and late complications provided they require a reoperation (i.e. reoperation for recurrences). Conversely, they are not designed to register medical variables, nor post-operative outcomes, what precludes detailed descriptions and multivariate analysis including such variables.

Registries capture a lot of different surgical variables, but their weakness lies on their perfectible completeness [38] of cases, data, and follow-up, despite methodological corrective measures such as those used in the CH registry, described in the method section. Thus, these two kinds of studies, administrative and registry-based, can be complementary in this type of research.

Hospital-level volume was found to be associated with fewer major complications in a large study by Chatta et al. [28]. They studied 54,075 patients operated on from ventral hernia at 2,049 hospitals. Patients treated at high-volume hospitals were less likely to experience a major complication (OR 0.88; 95% CI 0.82–0.96; p = 0.002) or wound-based complication (OR 0.84; 95% CI 0.76–0.92; p < 0.001). However, in terms of resource utilization, patients treated at high-volume hospitals were more likely to experience an extended length of stay (OR 1.14; 95% CI 1.09–1.12; p < 0.001) and an increase in costs (OR 1.23; 95% CI 1.17–1.29; p < 0.001) probably related to a more complex patient population.

Aquina et al. [29], analyzed reoperations for recurrent hernia over a 5-year period after open incisional hernia repairs in 18,047 patients. After adjusting for clinical factors, surgeons performing an average of > / = 36 repairs/year had significantly lower reoperation rates (HR = 0.59, 95% confidence interval (CI) = 0.48, 0.72), operative time (incidence rate ratio (IRR) = 0.67, 95% CI = 0.64, 0.71), and downstream charges (IRR = 0.63, 95% CI = 0.57, 0.69). Facility characteristics (volume, academic affiliation, location) were not associated with reoperation.

Annual volume was found to be significantly related to the risk of re-operation after laparoscopic hernia repair by Christophersen et al. [30]. They analyzed the risk of reoperation for recurrence after primary ventral hernia repair in 7,868 patients and found there was an increased risk of reoperation after laparoscopic umbilical or epigastric hernia repair for surgeons with ≤ 9 (hazard ratio 6.57; p = 0.008), 10 to 19 (hazard ratio 6.58; p = 0.011), and 20 to 29 (hazard ratio 13.59; p = 0.001) compared with > / = 30 cases/y. There were no differences in risk of reoperation after open mesh and open non-mesh repair in relation to annual surgeon volume.

An association was found between the annual case volume of the surgeon and the risk of medical complication in their patients. A possible explanation for this is that higher case volume surgeons work in multidisciplinary and skilled or specialized teams. Therefore, the risk for a medical complication can decrease. This protective effect is potentially not seen in surgical complications because high volume surgeons also operate on more difficult cases associated with poorer SSO/SSI and organ space complications.

The SSI and SSO rates are relatively low in our study. Club Hernie members are mainly experienced surgeons not only in parietal surgery, which explain their varying annual case volume, but in overall surgeries. Moreover, the complex cases are underrepresented in the club-Hernie cohort because most of the surgeons work in private sector and frequently refer their more difficult IVHR cases to public tertiary centers, which conversely have a very low participation or registration rate in the registry. We believe that this difference in the type of patients included in the database, is an explanation for the low number of reported SSI and SSO rates.

In the present study we focused on the relationship between surgeon annual case volume and intraoperative events, postoperative complications and ICU length of stay. In the two former studies the reoperation rate for hernia recurrence decreased after 30–36 cases performed per year. The annual surgeon volume categorization into 1–5, 6–50, 51–100, 101–125, > 125 cases, used in the present study was different from that used in Christophersen et al. [30] one. Moreover, we did not directly study the rate of reoperation for recurrence, because our endpoints were different but similarly, we observed a significant improvement regarding intra-operative complications, SSI, 30-day medical complications, and the duration of ICU stay. Our univariate analyses confirmed, comparatively with what found in the former studies, that high volume surgeons were less likely to suffer SSI, SSO, intra-operative complications and within 30 days medical complication. Nevertheless, no effect on patients reported hernia recurrence could be determined.

The relationships found in the univariate analyses, remain present in the multivariate analysie primarily for the C5 group. The C2 through C4 groups see the significant effect fade away after controlling for the confounding factors which might be an indication that the cases that they included in the hernia club database, are of less difficulty. The visual analysis showed that different roles of annual volume are present across the different types of ventral hernia repair: IVHR, PVHR and PSHR. As no effect can be noted in the PVHR group and a visual effect can be seen in the IVHR and PSHR groups, an indication that the annual ventral hernia repair volume of a surgeon should not be used as a whole but rather should be categorized into IVHR, PVHR, and PSHR is found. The results of this paper should primarily have implications for IVHR and PSHR, and less for PVHR as the effect is less prominent. The need for centralizing complex abdominal wall surgery is hereby highlighted.

### Limitations

Aside from a perfectible completeness of registries, discussed above, the present study entails some other limitations: among the titular CH members, the registered cases represent their actual activity. But for the temporary members (mainly found in the C1 subgroup) the registered cases do not perfectly match with their actual activity. A large part of their activity is not collected in the registry. When excluding the C1 subgroup the differences between other subgroups appeared slight and not statistically significant probably because of a homogeneous surgical cumulative experience across the titular CH members. Similar findings were reported among the Herniamed members [25]. The reoperation rate for recurrence was not included in the present study, replaced with patient-reported scar bulging which is far from a reliable measure for IH presence. A bulging without recurrence may be observed after complex cases which are mainly performed by high volume surgeons.

## Conclusion

In the present study on ventral hernia repairs, higher surgeon annual case volume was significantly related with less postoperative general complications such as surgical site infections, but not with less surgical site occurrences, nor with less patient-reported scar bulging. Other factors such as the type of repaired hernia in the surgeon case mix play an important role in a surgeon's outcomes.

## Supplementary Information

Below is the link to the electronic supplementary material.

Supplementary file1 (DOCX 268 KB)

## Data Availability

Data used in this study is available upon request from ‘Club Hernie’.
