# Supplementary material for: Is surgeon annual case volume related with intra and postoperative complications after ventral hernia repair? Uni- and multivariate analysis of prospective registry-based data
Source: Hernia. 2024 Aug 7;28(5):1935–44. doi: 10.1007/s10029-024-03129-2 (PMC11449983; doi:10.1007/s10029-024-03129-2)
Supplement: Supplementary file 1 — Supplementary file1 (DOCX 268 KB) [file 10029_2024_3129_MOESM1_ESM.docx]

Suppl. A: Characteristics of the study population
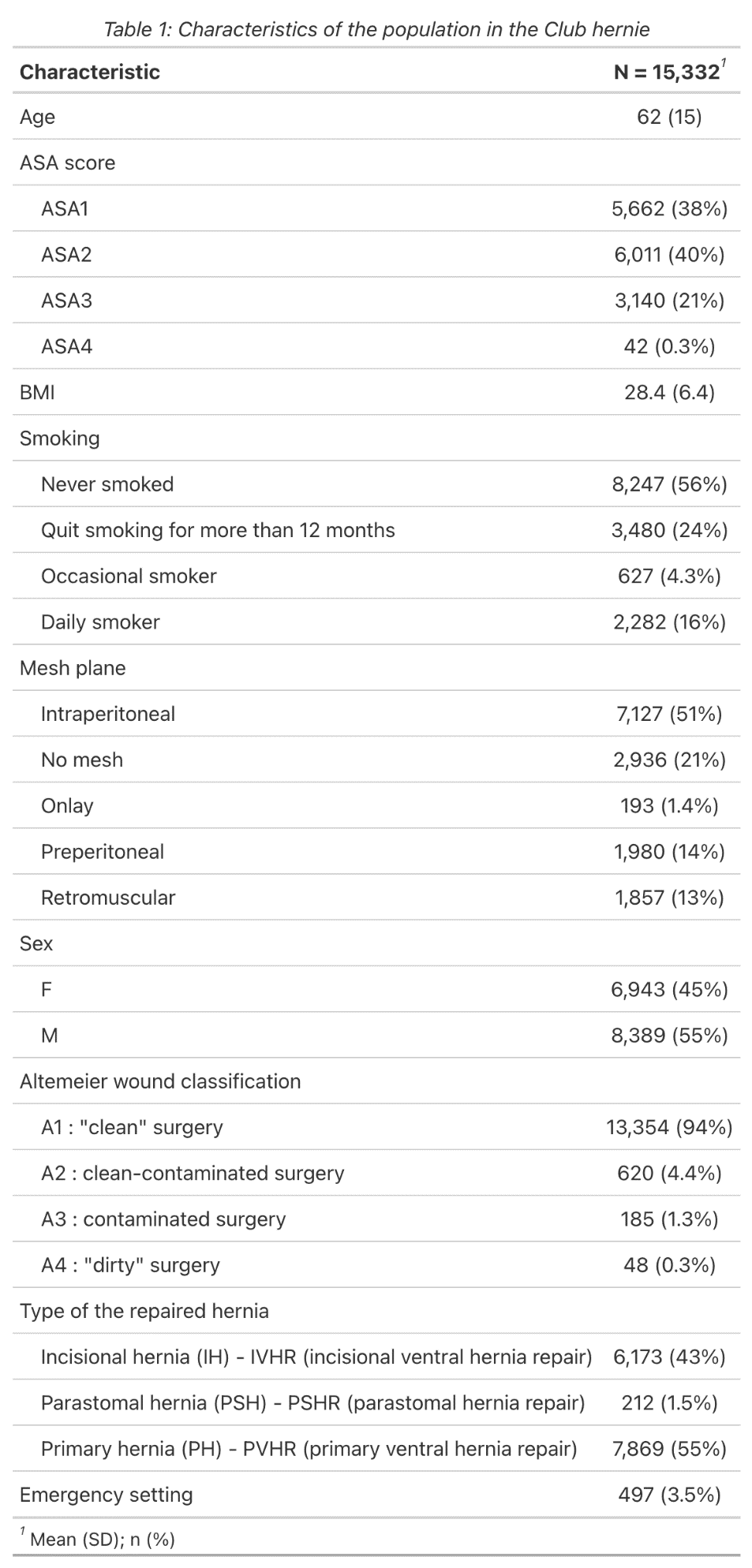


Suppl. B: Missing values in the population


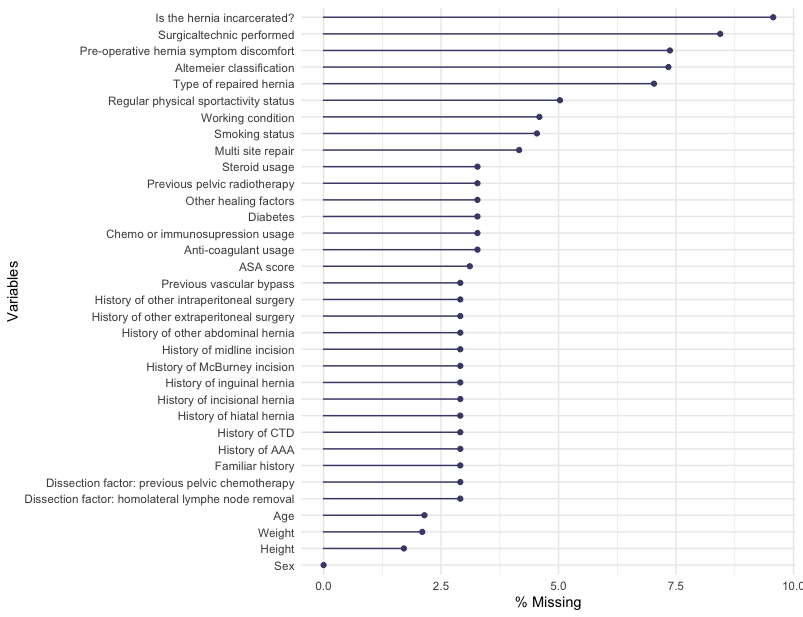


Suppl. C: Spread of the 199 studied surgeons according to their registered annual case volume

(each dot represents one surgeon, ordered from the lowest to the highest activity)


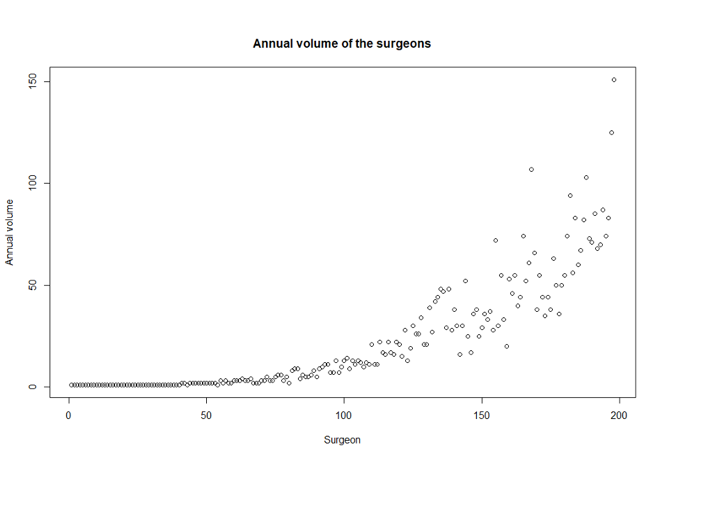


Surgeons

Suppl. D: Full table on regression analysis on ICU stay

| **Characteristic** | Table ICU Stay | | | |
| --- | --- | --- | --- | --- |
|  | **N** | **IRR***^1^* | **95% CI***^1^* | **p-value** |
| Peak annual volume^2^ |  |  |  |  |
| C1 | 114 | — | — |  |
| C2 | 2,514 | 0.76 | 0.65, 0.89 | **<0.001** |
| C3 | 4,322 | 0.78 | 0.66, 0.91 | **0.001** |
| C4 | 388 | 0.72 | 0.60, 0.86 | **<0.001** |
| C5 | 678 | 0.75 | 0.63, 0.89 | **<0.001** |
| Mesh plane |  |  |  |  |
| No mesh | 1,523 | — | — |  |
| Intraperitoneal | 3,820 | 1.00 | 0.94, 1.07 | >0.9 |
| Onlay | 122 | 0.90 | 0.75, 1.08 | 0.2 |
| Preperitoneal | 1,260 | 1.08 | 1.00, 1.16 | **0.048** |
| Retromuscular | 1,291 | 0.97 | 0.90, 1.05 | 0.5 |
| Component separation used? |  | 1.94 | 1.42, 2.65 | **<0.001** |
| Age | 8,016 | 1.00 | 1.00, 1.00 | 0.7 |
| Sex |  |  |  |  |
| F | 3,546 | — | — |  |
| M | 4,470 | 1.01 | 0.96, 1.05 | 0.8 |
| Type of repaired hernia |  |  |  |  |
| IVHR (incisional ventral hernia repair) | 3,758 | — | — |  |
| PSHR (parastomal hernia repair | 136 | 0.95 | 0.80, 1.14 | 0.6 |
| PVHR (primary ventral hernia repair) | 4,122 | 0.96 | 0.90, 1.03 | 0.2 |
| ASA score |  |  |  |  |
| ASA I | 3,049 | — | — |  |
| ASA II | 3,273 | 1.00 | 0.94, 1.05 | 0.9 |
| ASA III | 1,663 | 1.14 | 1.06, 1.22 | **<0.001** |
| ASA IV | 31 | 1.91 | 1.48, 2.46 | **<0.001** |
| BMI score | 8,016 | 1.00 | 1.00, 1.01 | 0.4 |
| Smoking |  |  |  |  |
| Never smoked | 4,490 | — | — |  |
| Quit smoking for more than 12 months | 1,982 | 0.97 | 0.92, 1.03 | 0.3 |
| Occasional smoker | 289 | 0.93 | 0.83, 1.05 | 0.3 |
| Daily smoker | 1,255 | 0.99 | 0.93, 1.05 | 0.7 |
| Altemeier wound score |  |  |  |  |
| A1 : "clean" surgery | 7,523 | — | — |  |
| A2 : clean-contaminated surgery | 343 | 1.38 | 1.26, 1.52 | **<0.001** |
| A3 : contaminated surgery | 115 | 1.67 | 1.45, 1.93 | **<0.001** |
| A4 : "dirty" surgery | 35 | 1.71 | 1.35, 2.15 | **<0.001** |
| Anti-coagulants usage |  | 1.05 | 0.99, 1.13 | 0.13 |
| Chemo therapy or immunosuppresion |  | 0.93 | 0.83, 1.03 | 0.2 |
| Pelvic radiotherapy |  | 0.94 | 0.78, 1.15 | 0.6 |
| Diabetes |  | 1.11 | 1.04, 1.19 | **0.001** |
| Steroid usage |  | 0.98 | 0.83, 1.15 | 0.8 |
| Other present healing factors |  | 0.97 | 0.86, 1.09 | 0.6 |
| Discomfort as pre-op hernia symptom |  | 1.00 | 0.95, 1.05 | >0.9 |
| History of abdominal aortic aneurysm |  | 1.16 | 0.85, 1.58 | 0.3 |
| History of CTD |  | 1.61 | 1.12, 2.32 | **0.010** |
| Familair history |  | 1.00 | 0.86, 1.16 | >0.9 |
| History of hiatal hernia |  | 0.91 | 0.74, 1.11 | 0.3 |
| Other relevent history present |  | 0.90 | 0.71, 1.14 | 0.4 |
| History of incisional hernia |  | 1.13 | 1.05, 1.22 | **0.001** |
| History of inguinal hernia |  | 0.96 | 0.90, 1.03 | 0.3 |
| History of midline hernia |  | 0.98 | 0.91, 1.05 | 0.6 |
| Dissection factor: McBurney |  | 0.97 | 0.90, 1.04 | 0.3 |
| Dissection factor: other extra-peritoneal surgery |  | 1.03 | 0.93, 1.14 | 0.6 |
| Dissection factor: other intra-peritoneal surgery |  | 1.03 | 0.97, 1.09 | 0.3 |
| Dissection factor: vascular bypass |  | 1.13 | 0.94, 1.35 | 0.2 |
| Dissection factor: homolateral node |  | 2.20 | 1.76, 2.74 | **<0.001** |
| Dissection factor: pelvic radiotherapy |  | 1.01 | 0.77, 1.33 | >0.9 |
| *^1^* IRR = Incidence Rate Ratio, CI = Confidence Interval  ^2^ peak annual volume was categorized into C1 (1-5 cases); C2 (6-50 cases); C3 (51-100 cases); C4 (101-125 cases); C5 (>125 cases). | | | | |

Suppl. E: Full table on regression analysis on combined intra and postoperative complications

| Regression analysis | | | | |
| --- | --- | --- | --- | --- |
| **Characteristic** | **Table combined intra or postoperative complications** | | | |
|  | **N** | **OR***^1^* | **95% CI***^1^* | **p-value** |
| Peak annual volume |  |  |  |  |
| C1 | 125 | — | — |  |
| C2 | 3,524 | 0.57 | 0.33, 0.96 | **0.036** |
| C3 | 6,869 | 0.39 | 0.23, 0.66 | **<0.001** |
| C4 | 960 | 0.52 | 0.29, 0.95 | **0.033** |
| C5 | 1,171 | 0.10 | 0.05, 0.21 | **<0.001** |
| Mesh plane |  |  |  |  |
| No mesh | 2,666 | — | — |  |
| Intraperitoneal | 6,132 | 1.03 | 0.78, 1.35 | 0.8 |
| Onlay | 182 | 1.00 | 0.56, 1.77 | >0.9 |
| Preperitoneal | 1,925 | 1.14 | 0.83, 1.56 | 0.4 |
| Retromuscular | 1,744 | 1.07 | 0.78, 1.46 | 0.7 |
| Component separation used? |  | 1.86 | 0.59, 5.86 | 0.3 |
| Age | 12,649 | 1.01 | 1.00, 1.02 | **<0.001** |
| Sex |  |  |  |  |
| F | 5,661 | — | — |  |
| M | 6,988 | 1.15 | 0.95, 1.39 | 0.2 |
| Type of repaired hernia |  |  |  |  |
| IVHR (incisional ventral hernia repair) | 5,526 | — | — |  |
| PSHR (parastomal hernia repair | 183 | 0.99 | 0.60, 1.62 | >0.9 |
| PVHR (primary ventral hernia repair) | 6,940 | 0.44 | 0.34, 0.57 | **<0.001** |
| ASA score |  |  |  |  |
| ASA I | 4,846 | — | — |  |
| ASA II | 5,139 | 1.31 | 1.01, 1.69 | **0.038** |
| ASA III | 2,623 | 1.92 | 1.44, 2.58 | **<0.001** |
| ASA IV | 41 | 4.12 | 1.84, 9.22 | **<0.001** |
| BMI score | 12,649 | 1.01 | 0.99, 1.02 | 0.3 |
| Smoking |  |  |  |  |
| Never smoked | 7,160 | — | — |  |
| Quit smoking for more than 12 months | 2,981 | 0.99 | 0.80, 1.23 | >0.9 |
| Occasional smoker | 529 | 1.39 | 0.91, 2.13 | 0.12 |
| Daily smoker | 1,979 | 1.04 | 0.80, 1.36 | 0.8 |
| Altemeier wound score |  |  |  |  |
| A1 : "clean" surgery | 11,895 | — | — |  |
| A2 : clean-contaminated surgery | 550 | 3.12 | 2.35, 4.15 | **<0.001** |
| A3 : contaminated surgery | 162 | 3.41 | 2.19, 5.32 | **<0.001** |
| A4 : "dirty" surgery | 42 | 3.69 | 1.65, 8.26 | **0.002** |
| Anti-coagulants usage |  | 1.41 | 1.12, 1.77 | **0.003** |
| Chemo therapy or immunosuppresion |  | 0.78 | 0.54, 1.12 | 0.2 |
| Pelvic radiotherapy |  | 0.72 | 0.37, 1.39 | 0.3 |
| Diabetes |  | 1.07 | 0.84, 1.36 | 0.6 |
| Steroid usage |  | 1.18 | 0.72, 1.90 | 0.5 |
| Other present healing factors |  | 1.02 | 0.70, 1.50 | >0.9 |
| Discomfort as pre-op hernia symptom |  | 1.18 | 0.96, 1.44 | 0.12 |
| History of abdominal aortic aneurysm |  | 1.83 | 0.74, 4.56 | 0.2 |
| History of CTD |  | 3.42 | 1.00, 11.7 | 0.050 |
| Familair history |  | 0.53 | 0.19, 1.47 | 0.2 |
| History of hiatal hernia |  | 0.82 | 0.41, 1.66 | 0.6 |
| Other relevent history present |  | 0.72 | 0.31, 1.66 | 0.4 |
| History of incisional hernia |  | 1.47 | 1.17, 1.86 | **0.001** |
| History of inguinal hernia |  | 0.90 | 0.68, 1.19 | 0.5 |
| History of midline hernia |  | 1.09 | 0.85, 1.41 | 0.5 |
| Dissection factor: McBurney |  | 0.99 | 0.74, 1.31 | >0.9 |
| Dissection factor: other extra-peritoneal surgery |  | 1.25 | 0.89, 1.76 | 0.2 |
| Dissection factor: other intra-peritoneal surgery |  | 1.31 | 1.06, 1.64 | **0.014** |
| Dissection factor: vascular bypass |  | 0.64 | 0.33, 1.24 | 0.2 |
| Dissection factor: homolateral node |  | 2.62 | 1.13, 6.11 | **0.025** |
| Dissection factor: pelvic radiotherapy |  | 1.40 | 0.62, 3.18 | 0.4 |
| ^1^ OR = Odds Ratio, CI = Confidence Interval | | | | |

*Suppl. G: Univariate logistic regression on the different outcomes for every annual volume group*

| Univariate Regression analysis | | | | | | | | | | | | | | | | | | | | |
| --- | --- | --- | --- | --- | --- | --- | --- | --- | --- | --- | --- | --- | --- | --- | --- | --- | --- | --- | --- | --- |
| Characteristic | **Table SSI** | | | | **Table SSO** | | | | **Table 30 Day Medical Complications** | | | | **Table Feel a Bulge** | | | | **Table Intra-Operative Complications** | | | |
|  | **N** | **OR***^1^* | **95% CI***^1^* | **p-value** | **N** | **OR***^1^* | **95% CI***^1^* | **p-value** | **N** | **OR***^1^* | **95% CI***^1^* | **p-value** | **N** | **OR***^1^* | **95% CI***^1^* | **p-value** | **N** | **OR***^1^* | **95% CI***^1^* | **p-value** |
| Peak-annual volume |  |  |  |  |  |  |  |  |  |  |  |  |  |  |  |  |  |  |  |  |
| C1 | 126 | — | — |  | 126 | — | — |  | 125 | — | — |  | 64 | — | — |  | 133 | — | — |  |
| C2 | 3,506 | 0.48 | 0.30 – 0.81 | 0.003 | 3,506 | 0.09 | 0.06 – 0.12 | <0.001 | 3,530 | 0.38 | 0.27 – 0.53 | <0.001 | 1,886 | 1.26 | 0.79 – 2.13 | 0.363 | 3,621 | 0.47 | 0.30 – 0.80 | 0.003 |
| C3 | 6,866 | 0.33 | 0.21 – 0.56 | <0.001 | 6,866 | 0.69 | 0.48 – 1.04 | 0.061 | 6,902 | 0.21 | 0.15 – 0.29 | <0.001 | 3,955 | 0.78 | 0.49 – 1.32 | 0.318 | 7,513 | 0.29 | 0.19 – 0.50 | <0.001 |
| C4 | 961 | 0.36 | 0.21 – 0.64 | <0.001 | 961 | 0.40 | 0.28 – 0.60 | <0.001 | 962 | 0.36 | 0.25 – 0.52 | <0.001 | 567 | 0.79 | 0.48 – 1.37 | 0.380 | 967 | 0.31 | 0.18 – 0.56 | <0.001 |
| C5 | 1,171 | 0.10 | 0.05 – 0.20 | <0.001 | 1,171 | 0.58 | 0.39 – 0.89 | 0.010 | 1,172 | 0.05 | 0.03 – 0.08 | <0.001 | 652 | 0.94 | 0.58 – 1.62 | 0.824 | 1,171 | 0.09 | 0.04 – 0.18 | <0.001 |
| *^1^* OR = Odds Ratio, CI = Confidence Interval | | | | | | | | | | | | | | | | | | | | |

*Suppl. H: Univariate negative-binomial regression on ICU stay for every annual volume group*

| *Characteristic* |  | *N* | *IRR^1^* | *95% CI^1^* | *p-value* |
| --- | --- | --- | --- | --- | --- |
| *Peak annual volume* |  |  |  |  |  |
| *C1* |  | *114* | *—* | *—* |  |
| *C2* |  | *2,514* | 1.50 | 1.37 – 1.63 | **<0.001** |
| *C3* |  | *4,322* | 0.75 | 0.68 – 0.82 | **<0.001** |
| *C4* |  | *388* | 0.75 | 0.69 – 0.82 | **<0.001** |
| *C5* |  | *678* | 0.67 | 0.61 – 0.75 | **<0.001** |
| *^1^ IRR = Incidence Rate Ratio, CI = Confidence Interval* |  |  |  |  |  |

*Suppl. I: Distribution of emergency surgery and incarcerated hernias throughout the annual volume groups*

| *Experience group ^1^* | *% of emergency cases* | *% Incarcerated hernias* |
| --- | --- | --- |
| *C1* | *3.8* | *22.8* |
| *C2* | *4.2* | *31.7* |
| *C3* | *3.0* | *28.8* |
| *C4* | *3.9* | *48.0* |
| *C5* | *4.0* | *38.0* |

*^1^ peak annual volume was categorized into C1 (1-5 cases); C2 (6-50 cases); C3 (51-100 cases); C4 (101-125 cases); C5 (>125 cases).*

Suppl. F: Full table on regression analysis on the binomial outcomes

| Regression analysis | | | | | | | | | | | | | | | | | | | | |
| --- | --- | --- | --- | --- | --- | --- | --- | --- | --- | --- | --- | --- | --- | --- | --- | --- | --- | --- | --- | --- |
| Characteristic | **Table SSI** | | | | **Table SSO** | | | | **Table 30 Day Medical Complications** | | | | **Table Feel a Bulge** | | | | **Table Intra-Operative Complications** | | | |
|  | **N** | **OR***^1^* | **95% CI***^1^* | **p-value** | **N** | **OR***^1^* | **95% CI***^1^* | **p-value** | **N** | **OR***^1^* | **95% CI***^1^* | **p-value** | **N** | **OR***^1^* | **95% CI***^1^* | **p-value** | **N** | **OR***^1^* | **95% CI***^1^* | **p-value** |
| Peak annual volume |  |  |  |  |  |  |  |  |  |  |  |  |  |  |  |  |  |  |  |  |
| C1 | 126 | — | — |  | 126 | — | — |  | 125 | — | — |  | 64 | — | — |  | 133 | — | — |  |
| C2 | 3,506 | 0.73 | 0.30, 1.77 | 0.5 | 3,506 | 1.00 | 0.50, 1.98 | >0.9 | 3,530 | 0.52 | 0.29, 0.93 | **0.029** | 1,886 | 1.47 | 0.61, 3.51 | 0.4 | 3,621 | 0.64 | 0.26, 1.55 | 0.3 |
| C3 | 6,866 | 0.63 | 0.26, 1.53 | 0.3 | 6,866 | 0.76 | 0.38, 1.50 | 0.4 | 6,902 | 0.34 | 0.19, 0.62 | **<0.001** | 3,955 | 1.02 | 0.42, 2.44 | >0.9 | 7,513 | 0.47 | 0.19, 1.13 | 0.091 |
| C4 | 961 | 0.60 | 0.22, 1.64 | 0.3 | 961 | 1.07 | 0.51, 2.24 | 0.9 | 962 | 0.51 | 0.26, 0.98 | **0.044** | 567 | 0.87 | 0.35, 2.22 | 0.8 | 967 | 0.53 | 0.19, 1.47 | 0.2 |
| C5 | 1,171 | 0.21 | 0.06, 0.71 | **0.012** | 1,171 | 0.72 | 0.34, 1.52 | 0.4 | 1,172 | 0.08 | 0.03, 0.19 | **<0.001** | 652 | 1.16 | 0.47, 2.89 | 0.7 | 1,171 | 0.15 | 0.04, 0.53 | **0.003** |
| Mesh plane |  |  |  |  |  |  |  |  |  |  |  |  |  |  |  |  |  |  |  |  |
| No mesh | 2,670 | — | — |  | 2,670 | — | — |  | 2,677 | — | — |  | 1,519 | — | — |  | 2,791 | — | — |  |
| Intraperitoneal | 6,116 | 0.87 | 0.55, 1.36 | 0.5 | 6,116 | 1.61 | 1.15, 2.25 | **0.005** | 6,155 | 1.02 | 0.74, 1.40 | >0.9 | 3,615 | 0.78 | 0.60, 1.01 | 0.058 | 6,681 | 1.04 | 0.67, 1.61 | 0.9 |
| Onlay | 182 | 3.89 | 1.98, 7.65 | **<0.001** | 182 | 5.08 | 2.98, 8.65 | **<0.001** | 183 | 0.65 | 0.31, 1.37 | 0.3 | 105 | 0.61 | 0.29, 1.28 | 0.2 | 191 | 1.49 | 0.66, 3.36 | 0.3 |
| Preperitoneal | 1,915 | 1.54 | 0.94, 2.53 | 0.087 | 1,915 | 2.12 | 1.46, 3.07 | **<0.001** | 1,925 | 1.24 | 0.86, 1.79 | 0.2 | 1,067 | 0.56 | 0.41, 0.78 | **<0.001** | 1,952 | 1.23 | 0.74, 2.05 | 0.4 |
| Retromuscular | 1,747 | 1.44 | 0.89, 2.34 | 0.14 | 1,747 | 2.71 | 1.88, 3.91 | **<0.001** | 1,751 | 1.12 | 0.78, 1.61 | 0.5 | 818 | 0.51 | 0.36, 0.73 | **<0.001** | 1,790 | 0.93 | 0.56, 1.54 | 0.8 |
| Component separation used? |  | 0.97 | 0.12, 7.73 | >0.9 |  | 1.78 | 0.51, 6.24 | 0.4 |  | 2.33 | 0.73, 7.44 | 0.2 |  | 0.59 | 0.07, 5.20 | 0.6 |  | 1.70 | 0.21, 13.7 | 0.6 |
| Age | 12,630 | 0.99 | 0.98, 1.00 | 0.10 | 12,630 | 0.99 | 0.99, 1.00 | 0.087 | 12,691 | 1.01 | 1.01, 1.02 | **<0.001** | 7,124 | 0.99 | 0.99, 1.00 | **0.048** | 13,405 | 1.00 | 0.99, 1.02 | 0.5 |
| Sex |  |  |  |  |  |  |  |  |  |  |  |  |  |  |  |  |  |  |  |  |
| F | 5,655 | — | — |  | 5,655 | — | — |  | 5,688 | — | — |  | 3,167 | — | — |  | 6,034 | — | — |  |
| M | 6,975 | 0.77 | 0.56, 1.05 | 0.10 | 6,975 | 1.20 | 0.98, 1.47 | 0.070 | 7,003 | 1.06 | 0.84, 1.32 | 0.6 | 3,957 | 0.91 | 0.75, 1.09 | 0.3 | 7,371 | 1.38 | 1.01, 1.89 | **0.044** |
| Type of repaired hernia |  |  |  |  |  |  |  |  |  |  |  |  |  |  |  |  |  |  |  |  |
| IVHR (incisional ventral hernia repair) | 5,519 | — | — |  | 5,519 | — | — |  | 5,541 | — | — |  | 3,128 | — | — |  | 5,829 | — | — |  |
| PSHR (parastomal hernia repair | 180 | 0.45 | 0.18, 1.10 | 0.078 | 180 | 0.32 | 0.13, 0.82 | **0.018** | 184 | 0.95 | 0.53, 1.69 | 0.9 | 85 | 1.73 | 0.85, 3.50 | 0.13 | 192 | 0.69 | 0.33, 1.43 | 0.3 |
| PVHR (primary ventral hernia repair) | 6,931 | 0.52 | 0.33, 0.81 | **0.004** | 6,931 | 0.76 | 0.59, 0.99 | **0.039** | 6,966 | 0.43 | 0.32, 0.58 | **<0.001** | 3,911 | 0.69 | 0.53, 0.88 | **0.003** | 7,384 | 0.46 | 0.29, 0.71 | **<0.001** |
| ASA score |  |  |  |  |  |  |  |  |  |  |  |  |  |  |  |  |  |  |  |  |
| ASA I | 4,847 | — | — |  | 4,847 | — | — |  | 4,866 | — | — |  | 2,783 | — | — |  | 5,127 | — | — |  |
| ASA II | 5,130 | 1.41 | 0.93, 2.14 | 0.11 | 5,130 | 1.39 | 1.08, 1.80 | **0.011** | 5,154 | 1.52 | 1.10, 2.09 | **0.010** | 2,901 | 1.07 | 0.86, 1.34 | 0.5 | 5,475 | 0.99 | 0.66, 1.48 | >0.9 |
| ASA III | 2,612 | 1.55 | 0.95, 2.52 | 0.080 | 2,612 | 1.56 | 1.15, 2.12 | **0.005** | 2,630 | 2.47 | 1.74, 3.52 | **<0.001** | 1,422 | 1.18 | 0.89, 1.58 | 0.3 | 2,760 | 1.02 | 0.63, 1.66 | >0.9 |
| ASA IV | 41 | 1.41 | 0.35, 5.58 | 0.6 | 41 | 1.45 | 0.41, 5.17 | 0.6 | 41 | 6.18 | 2.64, 14.5 | **<0.001** | 18 | 2.09 | 0.62, 7.11 | 0.2 | 43 | 1.55 | 0.40, 6.09 | 0.5 |
| BMI score | 12,630 | 1.01 | 1.0, 1.03 | 0.2 | 12,630 | 1.02 | 1.01, 1.04 | **<0.001** | 12,691 | 1.01 | 1.00, 1.02 | 0.2 | 7,124 | 1.00 | 0.99, 1.01 | >0.9 | 13,405 | 0.99 | 0.97, 1.02 | 0.6 |
| Smoking |  |  |  |  |  |  |  |  |  |  |  |  |  |  |  |  |  |  |  |  |
| Never smoked | 7,144 | — | — |  | 7,144 | — | — |  | 7,180 | — | — |  | 4,034 | — | — |  | 7,634 | — | — |  |
| Quit smoking for more than 12 months | 2,983 | 1.06 | 0.74, 1.52 | 0.8 | 2,983 | 0.96 | 0.76, 1.21 | 0.7 | 2,995 | 0.91 | 0.70, 1.19 | 0.5 | 1,641 | 1.09 | 0.87, 1.37 | 0.4 | 3,108 | 1.22 | 0.87, 1.72 | 0.3 |
| Occasional smoker | 530 | 0.26 | 0.06, 1.08 | 0.064 | 530 | 0.98 | 0.60, 1.59 | >0.9 | 532 | 1.68 | 1.06, 2.66 | **0.026** | 321 | 1.20 | 0.78, 1.85 | 0.4 | 547 | 0.75 | 0.26, 2.14 | 0.6 |
| Daily smoker | 1,973 | 1.23 | 0.82, 1.83 | 0.3 | 1,973 | 1.10 | 0.84, 1.42 | 0.5 | 1,984 | 1.10 | 0.80, 1.50 | 0.6 | 1,128 | 1.21 | 0.94, 1.54 | 0.13 | 2,116 | 0.95 | 0.61, 1.49 | 0.8 |
| Altemeier wound score |  |  |  |  |  |  |  |  |  |  |  |  |  |  |  |  |  |  |  |  |
| A1 : "clean" surgery | 11,885 | — | — |  | 11,885 | — | — |  | 11,939 | — | — |  | 6,697 | — | — |  | 12,627 | — | — |  |
| A2 : clean-contaminated surgery | 541 | 3.67 | 2.34, 5.75 | **<0.001** | 541 | 2.10 | 1.47, 3.01 | **<0.001** | 547 | 1.99 | 1.41, 2.83 | **<0.001** | 322 | 1.71 | 1.19, 2.47 | **0.004** | 569 | 6.85 | 4.57, 10.3 | **<0.001** |
| A3 : contaminated surgery | 162 | 9.68 | 5.62, 16.7 | **<0.001** | 162 | 1.73 | 0.91, 3.28 | 0.092 | 163 | 1.88 | 1.06, 3.35 | **0.032** | 75 | 1.73 | 0.86, 3.45 | 0.12 | 166 | 9.93 | 5.70, 17.3 | **<0.001** |
| A4 : "dirty" surgery | 42 | 12.6 | 5.30, 29.8 | **<0.001** | 42 | 1.46 | 0.44, 4.92 | 0.5 | 42 | 4.00 | 1.69, 9.49 | **0.002** | 30 | 2.52 | 1.01, 6.29 | **0.047** | 43 | 5.04 | 1.67, 15.2 | **0.004** |
| Anti-coagulants usage |  | 1.33 | 0.89, 1.97 | 0.2 |  | 1.30 | 1.00, 1.68 | 0.051 |  | 1.45 | 1.13, 1.88 | **0.004** |  | 1.01 | 0.76, 1.34 | >0.9 |  | 1.14 | 0.77, 1.70 | 0.5 |
| Chemo therapy or immunosuppresion |  | 1.04 | 0.59, 1.84 | 0.9 |  | 1.08 | 0.71, 1.65 | 0.7 |  | 0.73 | 0.48, 1.12 | 0.15 |  | 1.38 | 0.93, 2.04 | 0.11 |  | 0.96 | 0.53, 1.75 | >0.9 |
| Pelvic radiotherapy |  | 1.45 | 0.56, 3.74 | 0.4 |  | 0.51 | 0.18, 1.41 | 0.2 |  | 1.13 | 0.57, 2.24 | 0.7 |  | 0.72 | 0.31, 1.66 | 0.4 |  | 0.14 | 0.03, 0.70 | **0.016** |
| Diabetes |  | 1.15 | 0.78, 1.70 | 0.5 |  | 1.29 | 1.01, 1.66 | **0.044** |  | 1.08 | 0.83, 1.41 | 0.6 |  | 0.99 | 0.73, 1.34 | >0.9 |  | 1.38 | 0.94, 2.02 | 0.10 |
| Steroid usage |  | 1.36 | 0.64, 2.89 | 0.4 |  | 0.53 | 0.26, 1.07 | 0.077 |  | 1.53 | 0.92, 2.53 | 0.10 |  | 0.73 | 0.40, 1.33 | 0.3 |  | 0.65 | 0.24, 1.74 | 0.4 |
| Other present healing factors |  | 1.49 | 0.88, 2.53 | 0.14 |  | 1.02 | 0.65, 1.61 | >0.9 |  | 1.02 | 0.66, 1.59 | >0.9 |  | 1.22 | 0.73, 2.03 | 0.5 |  | 1.26 | 0.72, 2.22 | 0.4 |
| Discomfort as pre-op hernia symptom |  | 1.59 | 1.10, 2.31 | **0.015** |  | 1.17 | 0.94, 1.47 | 0.2 |  | 0.95 | 0.75, 1.19 | 0.7 |  | 1.30 | 1.05, 1.63 | **0.018** |  | 1.72 | 1.19, 2.49 | **0.004** |
| History of abdominal aortic aneurysm |  | 1.14 | 0.29, 4.52 | 0.9 |  | 1.81 | 0.71, 4.61 | 0.2 |  | 1.76 | 0.64, 4.83 | 0.3 |  | 2.84 | 0.96, 8.36 | 0.059 |  | 1.73 | 0.43, 7.04 | 0.4 |
| Familair history |  | 0.61 | 0.15, 2.54 | 0.5 |  | 0.89 | 0.41, 1.92 | 0.8 |  | 0.58 | 0.18, 1.87 | 0.4 |  | 0.64 | 0.32, 1.28 | 0.2 |  | 0.33 | 0.04, 2.53 | 0.3 |
| History of hiatal hernia |  | 1.30 | 0.50, 3.40 | 0.6 |  | 1.56 | 0.84, 2.90 | 0.2 |  | 0.92 | 0.42, 2.02 | 0.8 |  | 0.74 | 0.31, 1.75 | 0.5 |  | 0.49 | 0.12, 2.08 | 0.3 |
| Other relevent history present |  | 2.92 | 1.26, 6.76 | **0.012** |  | 0.80 | 0.28, 2.32 | 0.7 |  | 1.01 | 0.43, 2.36 | >0.9 |  | 1.25 | 0.51, 3.07 | 0.6 |  | - | - | - |
| History of incisional hernia |  | 1.27 | 0.87, 1.86 | 0.2 |  | 1.55 | 1.20, 2.01 | **<0.001** |  | 1.43 | 1.09, 1.87 | **0.010** |  | 1.74 | 1.33, 2.27 | **<0.001** |  | 1.68 | 1.16, 2.41 | **0.006** |
| History of inguinal hernia |  | 0.70 | 0.40, 1.19 | 0.2 |  | 0.93 | 0.69, 1.25 | 0.6 |  | 1.04 | 0.76, 1.41 | 0.8 |  | 1.26 | 0.96, 1.65 | 0.10 |  | 0.56 | 0.32, 0.98 | **0.040** |
| History of midline hernia |  | 0.84 | 0.55, 1.30 | 0.4 |  | 0.96 | 0.73, 1.27 | 0.8 |  | 1.09 | 0.82, 1.46 | 0.5 |  | 1.26 | 0.96, 1.65 | 0.091 |  | 1.05 | 0.69, 1.59 | 0.8 |
| Dissection factor: McBurney |  | 0.83 | 0.50, 1.38 | 0.5 |  | 0.92 | 0.67, 1.26 | 0.6 |  | 1.11 | 0.81, 1.53 | 0.5 |  | 0.97 | 0.73, 1.30 | 0.8 |  | 0.85 | 0.51, 1.43 | 0.5 |
| Dissection factor: other extra-peritoneal surgery |  | 1.09 | 0.62, 1.91 | 0.8 |  | 1.24 | 0.83, 1.84 | 0.3 |  | 1.11 | 0.75, 1.65 | 0.6 |  | 1.09 | 0.70, 1.70 | 0.7 |  | 1.64 | 0.97, 2.77 | 0.067 |
| Dissection factor: other intra-peritoneal surgery |  | 1.50 | 1.04, 2.16 | **0.030** |  | 1.40 | 1.11, 1.77 | **0.004** |  | 1.32 | 1.03, 1.70 | **0.031** |  | 1.09 | 0.87, 1.36 | 0.5 |  | 1.28 | 0.89, 1.85 | 0.2 |
| Dissection factor: vascular bypass |  | 2.53 | 1.13, 5.64 | **0.024** |  | 2.06 | 1.20, 3.54 | **0.009** |  | 0.77 | 0.38, 1.55 | 0.5 |  | 0.81 | 0.38, 1.76 | 0.6 |  | 0.83 | 0.28, 2.43 | 0.7 |
| Dissection factor: homolateral node |  | 1.54 | 0.37, 6.45 | 0.6 |  | 0.32 | 0.04, 2.44 | 0.3 |  | 2.66 | 1.05, 6.77 | **0.040** |  | 0.45 | 0.06, 3.61 | 0.5 |  | 1.54 | 0.40, 5.89 | 0.5 |
| Dissection factor: pelvic radiotherapy |  | 0.22 | 0.03, 1.93 | 0.2 |  | 1.33 | 0.41, 4.27 | 0.6 |  | 1.28 | 0.52, 3.15 | 0.6 |  | 0.35 | 0.08, 1.62 | 0.2 |  | 3.02 | 0.89, 10.3 | 0.077 |
| History of CTD |  |  |  |  |  | 3.11 | 0.87, 11.1 | 0.081 |  | 1.95 | 0.40, 9.56 | 0.4 |  | 2.54 | 0.66, 9.78 | 0.2 |  | 5.11 | 1.08, 24.1 | **0.039** |
| *^1^* OR = Odds Ratio, CI = Confidence Interval | | | | | | | | | | | | | | | | | | | | |
